# Motives for Exercising and Associations with Body Composition in Icelandic Adolescents

**DOI:** 10.3390/sports7060149

**Published:** 2019-06-20

**Authors:** Pablo Galan-Lopez, Francis Ries

**Affiliations:** Physical Education and Sports, Faculty of Educational Sciences, University of Seville (Research Lab HUM 962: Sports and Society), 41013 Sevilla, Spain; fries@us.es

**Keywords:** physical exercise, adolescents, motivation, Iceland

## Abstract

The aim of this study is to identify and analyze the motives that lead Icelandic teenagers to engage in physical exercise and the possible associations with variables of their body composition. For this purpose, the Self-Report of Reasons for the Practice of Physical Exercise questionnaire (AMPEF) was administered to 387 students (54% boys and 46% girls, M_age_ = 13.38 years) from Reykjavik (Iceland). The results reveal the subscales Revitalization and Enjoyment, Strength and Endurance, Challenge and Competition as the main motives for exercising among the participants. Boys score higher in all subscales than girls except for Revitalization and Enjoyment. Associations between the motive Weight Management and Appearance, and BMI and Fat % levels were found. It can be concluded that the participants’ physical exercise practice is based on the feelings and experiences they perceive in the sports practice process.

## 1. Introduction

According to data from the World Health Organization [1], obesity has increased from 7.4% to 17.0% in the last 25 years among the world population. Of every 10 children and adolescents between the ages of 2 and 17 years old, 20% are overweight and 10% are obese. Future forecasts predict that within two decades 140 million children (twice as many as today) will suffer from obesity. A recent survey conducted on a representative sample of the Icelandic population [2] showed that 66.7% of men and 50.4% of women aged between 18 and 80 years old were overweight. Several studies in Iceland have assessed overweight and obesity among adolescents and disturbing levels have been highlighted [3,4,5,6]. For example, among the Icelandic adolescent population, 13% are overweight and obese [7]. In 2008, 58.4% of the Icelandic population under the age of 20 were overweight and 23.3% were obese, with a prevalence of overweight of 65.1% for men and 51.7% for women [8].

As recent studies confirm the global prevalence of overweight and obesity [9], health promotion must take into account the perception, motivation and needs of the adolescent population [10,11]. Motivation is a vital component in reference to the initiation of sports practice and maintaining physical exercise [12,13]. The literature suggests that the most influential motivating elements seem to be friends, the coach, and school [14]. Therefore, it is crucial that physical education teachers make their students aware of the importance of physical activity and the adoption of a healthy lifestyle, as well as autonomously exercising on a regular basis [15]. 

Several studies have suggested that obese children and adolescents are more likely to become future obese adults [16,17]. Apart from the obvious physiological benefits, physical activity can also improve cognitive aspects (e.g., academic performance), as well as the mental health of children and adolescents [18]. Furthermore, the memory and the cognitive functions of the brain of physically active persons are increased [19,20].

Adolescents who practice sports regularly show higher levels of general and physical self-concept than those who practice irregularly or not at all [21]. Physical activity is directly related to a variety of psychological variables in women [22]. Hence, physically-active girls have a better self-concept and lower levels of anxiety and depression [23]. Thus, peer pressure, easy access to sport premises, initial orientation from a trainer, a variety of activities or team sports, the family’s physical activity participation and peer support and the feeling of satisfaction after weight loss have been identified as determinants of physical exercise [24,25].

Due to the lack of up-to-date scientific evidence in relation to the practice of physical exercise, its positive effects in the Icelandic adolescent population and its associations with motivation, the aim of this research was to analyze Icelandic adolescents’ motives for engaging in physical exercise and the possible associations with variables of their body composition.

## 2. Materials and Methods

The present study is a cross-sectional, descriptive and quantitative research that has followed the deontological standards recognized by the Declaration of Helsinki (revision of Hong-Kong in September 1989 and Edinburgh in 2000) and has been carried out in accordance with the recommendations of Good Clinical Practice of the EEC (document 111/3976/88 of July 1990). In addition, it was approved by the Icelandic National Bioethics Committee (Ref.: VSNb2017030026/03.01). 

### 2.1. Study and Sample Design

The participants in this study were school students aged 13–16 years old, living in Reykjavik (Iceland). Initially, 439 participants (204 girls and 235 boys) were recruited for the present study. Finally, a representative sample of 387 adolescents (178 girls (46%), M_age_ = 13.38 years old, SD = 1.14 and 209 boys (54%), M_age_ = 13.57 years old, SD = 1.13) completed all the measures, giving us a participation rate of 88.15%.

The students belonged to two different schools in the city of Reykjavik: Ölduselsskóli (45.8%) and Seljaskóli (54.2%). The sample size was calculated by applying a 95% confidence interval and an accuracy of 10% to the total adolescent school population in Reykjavik. The inclusion criteria for the present research were: participants (boys and girls) aged between 13 and 16 years old who had given an informed consent signed by their parents/tutors. In relation to their health status, the participants were those students who attended Physical Education classes regularly. The participants had no cognitive or physical/motor limitations. Adolescents were asked for verbal consent, being informed that their participation was voluntary and that they could withdraw from the research at any time.

### 2.2. Instruments

#### 2.2.1. AMPEF

The instrument used to assess the motives for exercising was the Icelandic translation of the “Auto-informe de Motivos para la Práctica de Ejercicio Físico“ (AMPEF, Self-Report of Reasons for the Practice of Physical Exercise), scale adapted to Spanish by Capdevila, Niñerola and Pintanel [26] from the Exercise Motivations Inventory-2 (EMI-2) [27], and, unlike the EMI-2, validated for an adolescent population. The questionnaire was provided to the participants with statements in English (from the original version) and in Icelandic. For the Icelandic version parallel back-translation was used [28,29,30]. 

The AMPEF scale consists of 48 items grouped into 11 subscales: Weight Management and Appearance, Revitalization and Enjoyment, Ill-Health Avoidance and Positive Health, Competition, Affiliation, Strength and Endurance, Social Recognition, Stress Management, Nimbleness, Challenge and Health Pressures (English and Icelandic versions of the questionnaire are provided as Supplementary Materials). The response format of the questionnaire is a Likert type from 0 (nothing true for me) to 10 (totally true for me). 

#### 2.2.2. Body Composition

The height of the subjects (barefoot) was measured with an accuracy of 0.1 cm using a portable height rod (Seca 213, Seca, Hamburg, Germany). The weight of the participants was measured with an accuracy of 0.10kg. The subjects wore light clothing. The body fat percentage of the subjects, as well as their weight, was registered via bioelectric impedance (Tanita Inner Scan BC-543, Tanita, Tokyo, Japan). The Body Mass Index (BMI) was calculated (with an accuracy of 0.01) from the relation of the body weight (kg) divided by the height squared of the subjects (m). The waist circumference was measured with a non-flexible anthropometric tape measure (Seca 201, Seca, Hamburg, Germany) with the subject standing and with an accuracy of 0.1 cm. The measurement point was the narrowest place of the space between the lowest rib and the upper iliac spine after normal expiration. For measurements of body composition parameters, the protocol marked on the ALPHA-Fitness Battery for measurement was followed at all times [31].

### 2.3. Data Analysis

Data for quantitative variables are presented as mean (M) + standard deviation (SD), while frequencies and percentages (%) have been used for qualitative variables. After checking the normality of the variables using the Kolmogorov-Smirnoff test, the T-Student test was used with the independent samples to carry out a comparison of the body composition variables with the different motivational subscales. To analyze girls’ and boys’ responses to different motivational subscales, descriptive analyses of the sample, calculation of means, standard deviations, frequency tables and scatter plots and reliability analysis were carried out. An ANOVA was carried out for gender and, in the case of significant differences being found, the post-hoc Bonferroni test was used. The level of statistical significance was set as p < 0.05. The statistical software IBM SPSS Statistics v.24 (SPSS Inc, Chicago, IL, USA) was used to carry out all the procedures mentioned above.

## 3. Results

The anthropometric characteristics of the participants are shown in Table 1. Although there are significant differences in the weight and height of the participants, no significant differences were found in relation to the BMI among boys and girls. Furthermore, boys show a lower percentage of fat than girls (p < 0.001), but a significantly larger waist circumference (p < 0.001). No significant differences were found in relation to the WHtR (Waist-to-height-ratio) among boys and girls.

Table 2 shows the reliability and internal consistency of the questionnaire used as well as the consistency of the 11 subscales and their average scores. The complete questionnaire showed a very high reliability index (α = 0.968) for the total sample. All the subscales showed α values above 0.800, except subscale 11 (Health Pressures). Analyzing the results at the global level (N = 387), the subscales with the highest scores are 6 (Strength and Endurance) and 2 (Revitalization and Enjoyment) followed by 10, 4, 5 and 3 (Challenge, Competition, Affiliation, Ill-health Avoidance, and Positive Health).

The score of each subscale was measured, differentiating according to the gender of the subjects. Of all the 11 subscales of the questionnaire, the girls only showed greater determination than the boys in subscale 8. In the rest of the questionnaire’s subscales, the boys had higher scores than girls for initiative/motivation for the practice of physical exercise. Subscale 2 has the highest scores for both girls and boys with almost the same results. This denotes that boys and girls exercise for the feelings of fun and wellbeing that this provides. Significant differences are observed in subscales 1, 4, 7 and 10 (*p* ≤ 0.05) with higher scores for boys. The motives with the highest scores for boys are subscales 2, 4, 6 and 10, whereas the girls’ motives for exercising correspond to subscales 2, 6, 9 and 5. (See Table 3).

**Figure 1:**
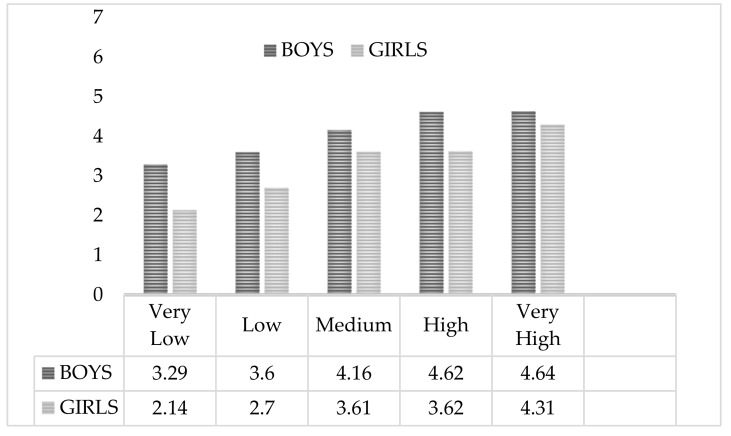
Weight Management and Appearance scoring by BMI based on the average levels established by Ortega et al. (2011) [32].

Analyzing the subscales of the questionnaire in relation to the parameters of body composition (BMI, fat % and waist circumference), an association between the values of BMI is observed. With increasing values of these parameters, the participants attribute more importance to subscale 1 (Weight Management and Appearance) (see Figure 1).

**Figure 2:**
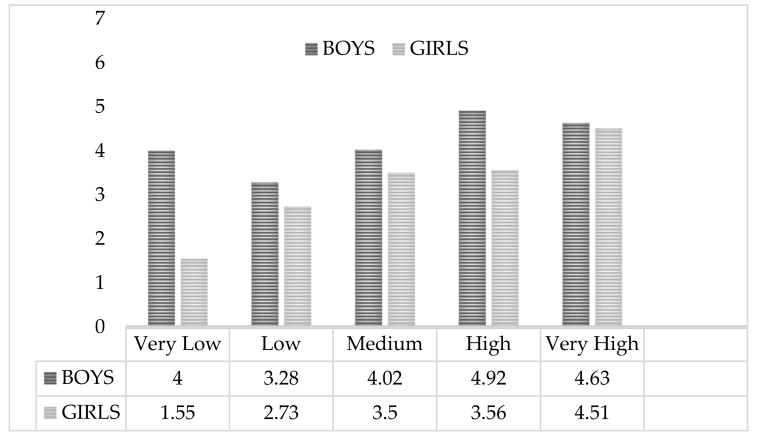
Weight Management and Appearance scoring by Fat % based on the average levels established by Ortega et al. (2011) [32].

In relation to the fat %, it is observed that in girls the score given to subscale 1 increases with the percentage of body fat, showing that girls value exercise more to improve their weight and body image as their body fat percentage increases (see Figure 2).

## 4. Discussion

The motives for exercising should be maintained lifelong because of the strong evidence concerning the beneficial effects of regular exercise [33]. Hence, it is vital to know what motivates adolescents to exercise as the highest rates for abandoning sports are reported at that age [34,35]. In this context, the present study is the first to analyze and describe the reasons why Icelandic teenagers exercise, as well as to associate them with their body composition characteristics. Significant differences were obtained between girls and boys in weight, height, % of body fat, and waist circumference (see Table 1). These results are comparable to those obtained in diverse studies on adolescent populations [36,37,38,39], in which girls had higher levels of adiposity, whereas boys showed higher weight, height, and waist circumference values. The weights, heights, and BMIs of the present study sample are comparable to the reference values provided by Ruiz et al. (2011) [32]. Unlike what was found by Wärnberg et al. (2006) [40], there is no prevalence of obesity in the adolescent participants of this study as the BMI, waist circumference, and % of body fat are considered medium values. Regarding the WHtR index values, these are analogous with the reference values provided by Ashwell et al. (2012) and Schneider et al. (2010) [41,42] and can be considered medium. 

The descriptive analysis of the questionnaire confirms that the motives which increase or maintain the practice of physical exercise are most of all Strength and Endurance, Revitalization and Enjoyment, Competition and Challenge. These results match those obtained in other research works [43,44]. Our study agrees with the previous work by Taylor et al. [45], which suggests that perceived competition is a powerful indicator of intentions to exercise: our work shows that competition is one of the most important determinants of physical exercise for the participants. The subscales that have the lowest impact on the practice or the maintaining of physical exercise are Health Pressures and Weight Management, and Appearance. Other studies conducted on adolescent populations have pointed out that if physically active people consider their physical abilities to be improvable, it is more likely that they will enjoy the activity and continue practicing physical exercise with the aim of progressing than if they believe that they have no options to progress [46,47,48].

Our work is also in agreement with the AVENA study results [44], which notes that participants (male and females) are more oriented toward attitudes related to the process of physical-sports activity than toward attitudes related to the result of this practice, our participants having valued more the Revitalization and Enjoyment, Competition and Challenge domains. Our results also match those presented by other studies, which highlighted fun and wellbeing as the greatest incentives when exercising [21,49]. In relation to the differences in motivation for boys and girls for practicing physical exercise, our study agrees with the conclusions of Jakobsen and Evjen, who found that intrinsic motives influenced sustained exercise in adolescents [50].

The subscale Weight Management and Appearance, historically attributed to the female gender, suggests a greater concern with respect to body image and aesthetics [51]. However, this research shows higher scores for boys, all in accordance with other authors, which corroborate that, at present, boys are increasingly more worried and concerned about their body image [52]. This trend is also supported by other authors as they found that adolescents in general (including boys) show a high concern for their body image [53,54]. These results are in contrast to those noted in another study by Montero et al. (2014), which concluded that appearance and weight were valued to a greater extent by the female participants [55]. It is possible that this is due to the influence of the aesthetic canon and current beauty which is justified in the study by Wilson and Rodgers [29].

Comparing the results concerning the participants’ body composition with diverse studies on the Icelandic adolescent population, our results differ from those obtained by Olafsdottir et al. [5] as the values of BMI, Fat % and waist circumference are mean scores. Regarding the associations between BMI and Fat % with the subscale Weight Management and Appearance, the results of our study agree with recent findings, since the participants with lower BMI and fat % practice exercise in order to have fun, feel better, compete and affiliate instead of for controlling their weight and improving their body image [56,57].

Due to the cross-sectional design of this study, only possible associations between the variables analyzed can be established. Despite its representative sample, the current research has an additional limitation. It is important to note that we relied exclusively on self-reported measures of the motives for exercising using the AMPEF questionnaire. 

## 5. Conclusions

The aim of this research was to analyze Icelandic adolescents’ motives for exercising as well as the potential associations with their body composition parameters. Our participants highlight as the main reasons behind their participation in physical exercise activities the experiences, feelings and emotions that they perceive during their practice. 

The current research also shows a change in the trend concerning body image and physical appearance as not only girls are concerned about it. Similarly, a positive attitude toward physical activity based primarily on appearance may be related to disturbing attitudes about eating and body image [58]. So, proper monitoring of the reasons that encourage adolescents to engage in physical exercise is essential. 

The weakest motives of the participants for practicing physical exercise are Health Pressures, Weight Management and Appearance, Social Recognition and Stress Management. That is why exercising for reasons of extrinsic motivations is discarded in the participants of our study. All things considered, the participants in the present study, exercise because they feel satisfaction, enjoy practice and feel better. Consequently, the practice of physical exercise is due to elements completely associated with intrinsic motivations.

The current research encourages physical education teachers to promote the practice of physical exercise among their adolescent students and to foster intrinsic motivation among them, thus supporting attitudes centered on the experiences and sensations perceived when exercising.

## Figures and Tables

**Table 1:** Anthropometric characteristics (N = 387).

Variables	Mean ± SD	Boys (n = 209)	Girls (n = 178)	*p*-Value
Age (years)	13.48 ± 1.14	13.57 ± 1.13	13.38 ± 1.14	0.097
Weight (kg)	57.29 ± 13.53	59.20 ± 14.21	55.05 ± 12.36	0.010*
Height (m)	1.64 ± 0.10	1.67 ± 0.11	1.61 ± 0.81	<0.001*
BMI (kg/m^2^)	22.26 ± 4.72	22.17 ± 4.91	22.37 ± 4.49	0.241
Body fat (%)	21.37 ± 8.79	17.37 ± 8.44	26.07 ± 6.61	<0.001*
Waist (cm)	72.05 ± 10.12	73.91 ± 10.41	69.86 ± 9.34	<0.001*
Waist to Height Ratio	0.44 ±0.06	0.44 ± 0.06	0.43 ± 0.05	0.142

Note: SD = Standard Deviation, BMI = Body Mass Index, Waist = Waist Circumference (p < 0.05). Reproduced with permission from [Galan-Lopez, P.; Ries, F.; Gisladottir, T.; Domínguez, R.; Sánchez-Oliver, A.J], [*Int. J. Environ. Res. Public Health*]; published by [MDPI], (2018).

**Table 2:** Internal consistency and total score of the AMPEF and its 11 subscales (N = 387).

SUBSCALES	CRONBACH ALPHA	MEAN (SD)
S1. Weight Management and Appearance	0.909	3.67 (2.40)
S2. Revitalization and Enjoyment	0.943	6.91 (2.82)
S3. Ill-Health Avoidance and Positive Health	0.833	5.56 (2.45)
S4. Competition	0.908	5.78 (3.29)
S5. Affiliation	0.836	5.57 (2.89)
S6. Strength and Endurance	0.879	5.97 (2.67)
S7. Social Recognition	0.807	4.17 (2.77)
S8. Stress Management	0.862	4.19 (3.02)
S9. Nimbleness	0.829	5.68 (2.83)
S10. Challenge	0.872	5.80 (2.75)
S11. Health Pressures	0.678	2.39 (2.50)
TOTAL (48 Items)	0.968	5.96 (1.89)

**Table 3:** AMPEF results by gender-related subscales (n = 387).

SUBSCALES	Boys (n = 209. M ± SD)	Girls (n = 178. M ± SD)	*p*-Value
S1. Weight Management and Appearance	3.99 ± 2.33	3.30 ± 2.43	0.004*
S2. Revitalization and Enjoyment	6.92 ± 2.86	6.91 ± 2.77	0.916
S3. Ill-Health Avoidance and Positive Health	5.76 ± 2.41	5.31 ± 2.47	0.068
S4. Competition	6.33 ± 3.25	5.13 ± 3.23	0.000*
S5. Affiliation	5.71 ± 2.91	5.40 ± 2.86	0.291
S6. Strength and Endurance	6.29 ± 2.64	5.60 ± 2.66	0.004*
S7. Social Recognition	4.84 ± 2.77	3.38 ± 2.56	0.000*
S8. Stress Management	3.92 ± 3.01	4.52 ± 2.99	0.048
S9. Nimbleness	5.89 ± 2.85	5.43 ± 2.80	0.114
S10. Challenge	6.23 ± 2.73	5.30 ± 2.70	0.001*
S11. Health Pressures	2.57 ± 2.64	2.17 ± 2.31	0.202

Note: M = Mean, SD = Standard Deviation (p < 0.05).

## Supplementary Materials

The following are available online at https://www.mdpi.com/2075-4663/7/6/149/s1.
