# Supplementary material for: Motives for Exercising and Associations with Body Composition in Icelandic Adolescents
Source: Sports (Basel). 2019 Jun 20;7(6):149. doi: 10.3390/sports7060149 (PMC6628225; doi:10.3390/sports7060149)
Supplement: Supplementary file 1 [file sports-07-00149-s001.pdf]

SELF-REPORT OF REASONS FOR THE PRACTICE OF PHYSICAL EXERCISE (AMPEF)

Á eftirfarandi síðum er að finna nokkrar fullyrðingar um það af hverju fólk stundar hreyfingu. Hvort sem þú stundar hreyfingu reglulega eða ekki skaltu lesa allar fullyrðingarnar vandlega og gefa til kynna, með því að gera hring um viðeigandi númer, hversu vel viðkomandi fullyrðing á við um þig eða ætti við um þig ef þú stundaðir hreyfingu reglulega. Ef þú telur að fullyrðing eigi alls ekki við um þig skaltu draga hring um „0“. Ef þú telur að fullyrðing eigi mjög við um þig skaltu draga hring um „10“. Ef þú telur fullyrðingu eiga við þig að hluta skaltu draga hring um 1-9 í samræmi við hversu vel þú telur hana endurspegla ástæður fyrir því hvers vegna þú stundar hreyfingu eða kynnir að stunda hreyfingu.

On the following pages are a number of statements concerning the reasons people often give when asked why they exercise. Whether you currently exercise regularly or not, please read each statement carefully and indicate, by circling the appropriate number, whether or not each statement is true for you personally, or would be true for you personally if you did exercise. If you do not consider a statement to be true for you at all, circle the ‘0’. If you think that a statement is very true for you indeed, circle the ‘10’. If you think that a statement is partly true for you, then circle 1-9 according to how strongly you feel that it reflects why you exercise or might exercise.

Mundu að við viljum vita hvers vegna þú velur að stunda hreyfingu eða kynnir að velja að stunda hreyfingu, ekki hvort þú telur að fullyrðingarnar séu góðar ástæður fyrir einhvern til að stunda hreyfingu.

Remember, we want to know why you personally choose to exercise or might choose to exercise, not whether you think the statements are good reasons for anybody to exercise.

SELF-REPORT OF REASONS FOR THE PRACTICE OF PHYSICAL EXERCISE (AMPEF)											
**Ég stunda hreyfingu (eða kynni að stunda hreyfingu) // Personally, I exercise (or might exercise)**	**Á alls ekki við um mig // Not at all true for me**							**Á mjög við um mig // Very true for me**			
1. Til að halda mér grannri/grönnum // To stay slim	0	1	2	3	4	5	6	7	8	9	10
2. Til að forðast veikindi // To avoid il-health	0	1	2	3	4	5	6	7	8	9	10
3. Hreyfing veitir mér vellíðan // Because it makes me feel good	0	1	2	3	4	5	6	7	8	9	10
4. Til að sýna öðrum hvaða eiginleikum ég bý yfir // To show my worth to others	0	1	2	3	4	5	6	7	8	9	10
5. Til að auka líkamlegt heilbrigði // To have a healthy body	0	1	2	3	4	5	6	7	8	9	10
6. Til að styrkja mig // To build up my strength	0	1	2	3	4	5	6	7	8	9	10
7. Það veitir mér vellíðan að leggja mig fram // Because I enjoy the feeling of exerciting myself	0	1	2	3	4	5	6	7	8	9	10
8. Til að verja tíma með vinum // To spend time with friends	0	1	2	3	4	5	6	7	8	9	10
9. Því læknirinn minn ráðlagði mér að hreyfa mig //Because my doctor advised me to exercise	0	1	2	3	4	5	6	7	8	9	10

10. Því mér finnst gaman að reyna að sigra í íþróttum // Because I like trying to win in physical activities	0	1	2	3	4	5	6	7	8	9	10
11. Til að halda mér og /eða verða snarpari og liprari // To stay/become more agile	0	1	2	3	4	5	6	7	8	9	10
12. Til að vera með markmið til að stefna að // To give me goals to work towards	0	1	2	3	4	5	6	7	8	9	10
13. Til að léttast // To lose weight	0	1	2	3	4	5	6	7	8	9	10
14. Til að koma í veg fyrir heilsufarsvandamál // To prevent health problems	0	1	2	3	4	5	6	7	8	9	10
15. Því mér finnst íþróttaiðkun hressandi // Because I find exercise invigorating	0	1	2	3	4	5	6	7	8	9	10
16. Til að líta vel út // To have a good body	0	1	2	3	4	5	6	7	8	9	10
17. Til að bera líkamlega getu mína saman við getu annarra // To compare my abilities with other peoples	0	1	2	3	4	5	6	7	8	9	10
18. Því það hjálpar mér við að losa um spennu // Because it helps to reduce tension	0	1	2	3	4	5	6	7	8	9	10
19. Því ég vil viðhalda heilbrigði / Because I want to maintain good health	0	1	2	3	4	5	6	7	8	9	10
20. Til að auka úthald // To increase my endurance	0	1	2	3	4	5	6	7	8	9	10
21. Því æfingar veita mér ánægju // Because I find exercising satisfying in and of itself	0	1	2	3	4	5	6	7	8	9	10
22. Til að njóta félagslegra áhrifa íþróttaiðkunar // To enjoy the social aspects of exercising	0	1	2	3	4	5	6	7	8	9	10
23. Til að koma í veg fyrir ættgengan sjúkdóm // To help prevent and illness that runs in my family	0	1	2	3	4	5	6	7	8	9	10
24. Því mér finnst gaman að keppa // Because I enjoy competing	0	1	2	3	4	5	6	7	8	9	10
25. Til að viðhalda liðleika // To maintain flexibility	0	1	2	3	4	5	6	7	8	9	10
26. Til að mæta persónulegum áskorunum // To give me personal challenges to face	0	1	2	3	4	5	6	7	8	9	10
27. Til að hjálpa mér við þyngdarstjórnun // To help control my weight	0	1	2	3	4	5	6	7	8	9	10
28. Til að koma í veg fyrir hjartasjúkdóma // To avoid heart disease	0	1	2	3	4	5	6	7	8	9	10
29. Til að bæta útlit mitt // To improve my appearance	0	1	2	3	4	5	6	7	8	9	10

30. Til að öðlast viðurkenningu fyrir það sem ég hef áorkað // To gain recognition for my accomplishments	0	1	2	3	4	5	6	7	8	9	10
31. Til að hjálpa við streitustjórnun // To help manage stress	0	1	2	3	4	5	6	7	8	9	10
32. Til að mér finnist ég heilbrigðari // To feel more healthy	0	1	2	3	4	5	6	7	8	9	10
33. Til að verða sterkari // To get stronger	0	1	2	3	4	5	6	7	8	9	10
34. Til að njóta upplifunarinnar af íþróttaiðkuninni // For enjoyment of the experience of exercising	0	1	2	3	4	5	6	7	8	9	10
35. Til að skemmta mér í hreyfingu með öðru fólki // To have fun being active with other people	0	1	2	3	4	5	6	7	8	9	10
36. Til að hjálpa mér við að ná mér af veikindum/meiðslum // To help recover from illness/injury	0	1	2	3	4	5	6	7	8	9	10
37. Því ég hef gaman af líkamlegum keppnum // Because I enjoy physical competition	0	1	2	3	4	5	6	7	8	9	10
38. Til að auka og/eða viðhalda liðleika // To stay/become flexible	0	1	2	3	4	5	6	7	8	9	10
39. Til að þróa með mér persónulega færni // To develop personal skills	0	1	2	3	4	5	6	7	8	9	10
40. Því æfingar hjálpa mér við að brenna hitaeiningum // Because exercise helps me to burn calories	0	1	2	3	4	5	6	7	8	9	10
41. Til að vera meira aðlaðandi // To look more attractive	0	1	2	3	4	5	6	7	8	9	10
42. Til að gera hluti sem aðrir geta ekki // To accomplish things that others are incapable of	0	1	2	3	4	5	6	7	8	9	10
43. Til að losa um spennu // To release tension	0	1	2	3	4	5	6	7	8	9	10
44. Til að byggja upp vöðva // To develop my muscles	0	1	2	3	4	5	6	7	8	9	10
45. Því mér líður best þegar ég æfi // Because I feel at my best when exercising	0	1	2	3	4	5	6	7	8	9	10
46. Til að eignast nýja vini // To make new friends	0	1	2	3	4	5	6	7	8	9	10
47. Því mér finnst líkamleg áreynsla skemmtileg, einkum þegar keppni kemur við sögu // Because I find physical activities fun, especially when competition is involved	0	1	2	3	4	5	6	7	8	9	10
48. Til að meta hvort ég hef náð markmiðum mínum // To measure myself against personal standars	0	1	2	3	4	5	6	7	8	9	10
